# Interpreting mismatches between linguistic and genetic patterns among speakers of Tanimuka (Eastern Tukanoan) and Yukuna (Arawakan)

**DOI:** 10.1098/rsfs.2022.0056

**Published:** 2022-12-09

**Authors:** Leonardo Arias, Nicholas Q. Emlen, Sietze Norder, Nora Julmi, Magdalena Lemus Serrano, Thiago Chacon, Jurriaan Wiegertjes, Austin Howard, Matheus C. B. C. Azevedo, Allison Caine, Saskia Dunn, Mark Stoneking, Rik Van Gijn

**Affiliations:** ^1^ Leiden University Centre for Linguistics, Leiden, The Netherlands; ^2^ Department of Evolutionary Genetics, Max-Planck-Institute for Evolutionary Anthropology, Leipzig, Germany; ^3^ University of Groningen (Campus Fryslân), Groningen, The Netherlands; ^4^ Copernicus Institute of Sustainable Development, Environmental Science Group, Utrecht University, Princetonlaan 8a, 3584 CB Utrecht, The Netherlands; ^5^ University Aix-Marseille, Marseille, France; ^6^ Universidade de Brasília, Brasília, Brazil; ^7^ University of Wyoming, Laramie, WY, USA; ^8^ Laboratoire de Biométrie et Biologie Evolutive, Université Lyon 1, CNRS, UMR 5558, Villeurbanne, France

**Keywords:** genetic admixture, language contact, language change, human population history, ethnography, Amazonia

## Abstract

Northwestern Amazonia is home to a great degree of linguistic diversity, and the human societies in that region are part of complex networks of interaction that predate the arrival of Europeans. This study investigates the population and language contact dynamics between two languages found within this region, Yukuna and Tanimuka, which belong to the Arawakan and Tukanoan language families, respectively. We use evidence from linguistics, ethnohistory, ethnography and population genetics to provide new insights into the contact dynamics between these and other human groups in NWA. Our results show that the interaction between these groups intensified in the last 500 years, to the point that it is difficult to differentiate between them genetically. However, this close interaction has led to more substantial contact-induced language changes in Tanimuka than in Yukuna, consistent with a scenario of language shift and asymmetrical power relations.

## 1. Introduction

Northwestern Amazonia (NWA), comprising the totality of Colombian Amazonia and border areas between Colombia, Venezuela, Brazil and Peru, is an area of great linguistic and cultural diversity [1,2]. This area also exhibits high ecological heterogeneity, including seasonally flooded savannahs to the north, the Andean foothills to the west and northwest, the westernmost ridge of the Guiana shield to the east, and Amazonian rainforest. This landscape heterogeneity is amplified by a complex network of rivers that drains the area into the Orinoco and Amazon Rivers. In this complex topography there coexists a panoply of ethnolinguistic groups belonging to the Arawakan, Tukanoan, Cariban, Tupian, Nadahup and Kakua-Nukak languages families, in addition to several language isolates or near-isolates. These groups exhibit a diverse set of subsistence practices, cosmologies, rituals, postmarital residence patterns and marriage practices. Together, this diversity is contained within a large sociocultural complex, in which many groups share some elements of their social organization, ritual cycles of exchange and several elements of the narratives about their mythical origins [3].

At the centre of this large area, social exogamy and multilingualism create a marriage practice known as linguistic exogamy, in which marriages are forged between members of different patrilineal descent groups which are associated with different languages. These marriages bring together speakers of several Eastern Tukanoan (ET) and Arawakan languages, as well as the Carijona language of the Cariban family (with Nadahup languages playing a more peripheral role) [3–7]. Linguists have long noted the impacts of this social structure on the languages of the area, including structural convergence but not widespread lexical borrowing, since the lexicons serve the function of marking patrilineal descent group membership [1,5,8].

This paper examines the population and language contact dynamics between two languages found within this region, Yukuna and Tanimuka, which belong to the Arawakan and Tukanoan language families, respectively. Yukuna is the identity language of the Yukuna and Matapi groups, who number approximately 700–1000 people [9]; Tanimuka is the identity language of the Tanimuka and Letuama groups, with a combined ethnic population of approximately 500 people [10]. These ethnolinguistic groups coexist in various communities along the Mirití-Parana River. In addition to many shared cultural practices, the languages of the Tanimuka and Yukuna have undergone notable mutual contact-induced linguistic changes [5,11,12]. A recent genetic study [13] found, furthermore, that the two social units share identical mitochondrial genomes, resulting from extensive intermarriage mainly involving the exchange of women. Furthermore, Franky [14] has proposed that the Tanimuka have an Arawakan origin, based on an analysis of their ethnography and oral histories; this would suggest a scenario in which the Tanimuka are a formerly Arawakan-speaking group which shifted to a Tukanoan language. As we discuss below, this hypothesis is the subject of the current paper.

Given that contact and admixture among human populations has been a constant throughout history [15,16], globally there are several examples that have used genetic data to resolve potential cases of language/cultural shift and to understand the relationship between genetic and linguistic evolution [17–25]. However, there are relatively few examples that tell us what to expect in situations of extensive contact regarding the language, genetic structure and cultural identity of groups. In the case of language shift, in which a group gives up their original language to adopt a new language and perhaps a sociocultural identity too, it is the shifting group that induces changes in their version of the target language. Thomason & Kaufman [26] have suggested that language shift induces both phonological and syntactic changes. Ross [27] has suggested that phonological transfer, constructional calquing, transfer of specialized vocabulary and simplification are expected. By contrast, convergence due to extensive contact is expected to induce lexical and grammatical calquing, as well as syntactic restructuring and complexification [27,28] (see §3.2 and particularly Fig. 6 for more information). Furthermore, it is widely acknowledged that the dynamics of contact-induced language change are always determined by sociocultural factors, such as power relations between groups and individuals' attitudes and ideologies toward the languages of the others [5,6,28,29].

Extensive contact in the context of intermarriage can be expected to lead to different genetic patterns depending on sociocultural factors such as marriage practices and postmarital residence rules. For instance, in the absence of asymmetrical power relations or strong rules for endogamous versus exogamous marriage among the groups in contact, one would expect to find relatively few genetic differences. However, it has been shown that among small-scale human societies, intermarriage is often sex-biased, with a preference toward one of the sexes marrying into the other group. Since patrilocality is a frequent postmarital residence pattern among human societies, it is common to find women moving to the husband's ancestral territory [30–32]. Furthermore, it has been observed that in areas where farmers have a dominant position over foragers, genetic admixture is often sex-biased involving farmer males and forager females [33–35]. More specifically, a higher movement of women among groups reduces population differentiation on the maternally inherited mitochondrial DNA (mtDNA) and patrilocality will lead to an increase in population differentiation on the paternally inherited Y-chromosome (MSY) genetic variation. At the nuclear DNA level, extensive contact and genetic admixture between ethnolinguistically differentiated groups will lead to mismatches between genetic and linguistic affiliation, as these groups will look genetically more similar among them than to their linguistic relatives. In addition, in cases of sex-biased admixture comparisons between X-chromosome and autosomal genetic variation have been traditionally used to study sex-biased demography [36]. However, in cases of complex and extensive contact such methods encounter problems to resolve the process and timescale of admixture, highlighting the complex interplay between demographic and cultural processes in determining patterns of autosomal genetic variation [37].

In this study, we offer novel insights into the population history and the dynamics of language change in NWA by bringing together evidence from linguistics, ethnohistory, ethnography and population genetics regarding Yukuna and Tanimuka, as well as their place within the broader NWA social panorama. Of course, there are important differences in the way culture, language and genes are transmitted and changed, and differences in the time scales on which these processes operate (for discussion see [38–40]). Therefore, one should not expect to find perfect matches between culture, language, and genes. However, if we consider those types of data together, each can inform different aspects of human history that otherwise would be misinterpreted if based on the insights from a single disciplinary approach. Furthermore, we expect that at a local scale, mismatches between sociocultural patterns, language families, language contact dynamics, and genetic admixture could be more easily dissected and interpreted than at continental scales. Thus, we use this multidisciplinary approach to assess which of two scenarios is more likely: first, that Tanimuka speakers descend from an Arawakan group related to Yukuna, which later adopted an Eastern-Tukanoan (ET) language; or second, that Tanimuka speakers descend from an ET speaking group, but that extensive contact and intermarriage with Yukuna resulted in notable convergences in their language and culture.

## 2. Material and methods

To learn more about the contact situation between these groups, we adopt a multidisciplinary approach involving linguistic, sociocultural and genetic databases. Each of these types of data informs us about different sociocultural, demographic and historical aspects of the human societies of Northwestern Amazonia. Here we describe the kinds of data and the analytical approaches used for each type of evidence.

### 2.1. Genetic data

We collected previously reported genetic data from several indigenous groups from NWA (table 1), including uniparental [13,31] and newly generated genome-wide SNP data genotyped on the Affymetrix Human Origins Array. For the genome-wide SNP data, we restricted our analyses to only autosomal SNPs, markers present in chromosomes 1–22, excluding all other markers outside these chromosomes. In addition, we excluded SNP positions (loci) and individuals with more than 10% missing calls. After these filterings we were left with 5 77 323 SNPs. The uniparental data includes complete mitochondrial genome sequences (mtDNA) and sequences of a region of 2.3 mega bases of the male-specific Y-chromosome (MSY), which allow us to distinguish between male and female population histories.

**Table 1.** Ethnolinguistic sample, including sources of the linguistic data and number of individuals for each genetic marker.

language name	glottocode	affiliation	linguistic source(s)^a^	MSY (*n*)^b^	mtDNA (*n*)	nuclear data (*n*)
Achagua	acha1250	Arawakan	[41]	5	6	5
Barasana	bara1380	Tukanoan	[42–44]	2	4	2
Carapana	cara1272	Tukanoan	[45]	1	1	1
Kakua	cacu1241	Kakua-Nukak	[46]	0	0	0
Koreguaje	kore1283	Tukanoan	[47]	12	12	10
Kubeo	cube1242	Tukanoan	[48,49]	3	5	4
Desano	desa1247	Tukanoan	[50,51]	14	17	14
Warekena	guar1293	Arawakan	[52]	0	0	0
Kotiria	guan1269	Tukanoan	[53]	5	5	5
Hup	hupd1244	Naduhup	[54]	0	0	0
Piapoco	piap1246	Arawakan	[55]	17	18	18
Resígaro	resi1247	Arawakan	[56]	0	0	0
Sekoya	seco1241	Tukanoan	[57]	0	0	0
Siona	sion1247	Tukanoan	[58]	10	17	17
Tanimuka	tani1257	Tukanoan	[59]	4	10	6
Tariana	tari1256	Arawakan	[60]	0	0	0
Tukano	tuca1252	Tukanoan	[61,62]	2	8	6
Yukuna	yucu1253	Arawakan	[63]	18	31	18
Matapi	yucu1253	Arawak	Not included	6	8	6
Nukak	nuka1242	Kakua-Nukak	Not included	11	16	10
Curripaco	curr1243	Arawak	Not included	13	16	6

^a^Linguistic sources are described in electronic supplementary material.

^b^*n* refers to the number of individuals included.

We used two analytical approaches with the genome-wide data. The first one uses allele-frequency-based methods and includes PCA (as implemented in the Eigensoft package [64]), model-based ancestry estimation with ADMIXTURE [65], and f-statistics [16,66]. For PCA and ADMIXTURE we merged the NWA dataset with data from other modern Native American populations previously described [16,67–69], using the program mergeit implemented in EIGENSOFT software package v. 7.2.0 with default settings [64]. For the ADMIXTURE analysis, we pruned the merged dataset for linkage disequilibrium with PLINK v. 1.90b5.2, using the command –indep-pairwise 100 20 0.5 leaving a total of 87 297 SNPs. ADMIXTURE clusters together genetically similar individuals, based on the estimation of allele frequencies, and assigns ancestry components from an *a priori*-defined number of ‘K’ ancestral source populations [65]. We ran ADMIXTURE from *K* = 2 to *K* = 14 with 20 replicates for each K and used the cross-validation error procedure to find the ‘*K*’ that best describes the genotype data. We visualized ADMIXTURE results with the software pong [70].

We used ADMIXTOOLS [16] as implemented in the R package admixr [71] and the R package ADMIXTOOLS2 [72] to estimate pairwise f_2_-statistics, outgroup-f_3_-statistics, f_4_-statistics and f4-ratios (see [66] for discussion). These statistics are useful to infer genetic relationships and to test hypotheses about genetic admixture and common ancestry among populations.

The second approach uses haplotype-based methods, that is, long and uninterrupted blocks of DNA inherited from a set of common ancestors [73]. We performed statistical phasing of the genotype data with the software SHAPEIT v. 2.r904 [74], using the recombination map and a reference panel of American populations from the 1000 Genomes Project Phase 3 (1000 Genomes Project Consortium *et al*. 2015). We ran SHAPEIT with options –burn 10, –prune 10 and –main 30 for iteration number with 500 conditioning states, leaving other parameters as default as previously reported [20]. We used the phased output to run the software ChromoPainterv2 [75], which reconstructs the haplotype(s) of a ‘recipient’ individual using the haplotypes from all other individuals in the sample as potential donors. This process is repeated for every haplotype in turn, so every individual's genome is ultimately reconstructed in terms of all the other individual genomes [75]. In addition, we used the software RefinedIBD to detect identity-by-descent (IBD) and homozygous-by-descent (HBD) blocks, IBD and HBD were merged and split by length category into three datasets as follows: 1–5 centimorgans (cM), 5–10 cM and over 10 cM, as previously described [19,20]. The plot of sharing of IBD was adapted using scripts described here: https://github.com/dangliu/Massim_project.

With the uniparental data we focused on reconstructing haplotype networks, implemented in the R-package pegas v. 1.1 [76], using the minimum spanning network method. Haplotype network visualizations are useful to infer phylogenetic relationships among individuals' haplotypes and to identify the sharing of identical haplotypes among groups, which are informative about recent common ancestry or sex-specific genetic admixture.

### 2.2. Sociocultural data

We started by compiling a large set of sociocultural data from ethnolinguistic groups in Western South America (see electronic supplementary material, data availability section), designed to address a broad range of questions regarding the population history of this part of the continent. We subset this database to match the ethnolinguistic groups described in table 1, leaving us 117 variables from the 18 societies. The dataset covers the most basic types of social and cultural information available in the ethnographic literature, including subsistence practices, settlements and architecture, kinship and marriage, social and political organization, material culture, body modification, cosmology and ritual. The data come from monographs, articles and book chapters, travel reports, pre-existing datasets, personal communication with experts and a range of other ethnographic materials. A detailed list of sources and a description of our strategy for coding variables can be found in the electronic supplementary material. A major limitation in developing a dataset like this is the patchy and inconsistent ethnographic record of Western Amazonia, which inevitably leads to a fair amount of missing data. We followed the best practices in handling missing data [77].

For this paper, given the importance of gender-specific sociocultural practices in NWA, we chose to separately analyse practices generally transmitted from fathers to sons, and those generally transmitted from mothers to daughters (table 2). (This means that these gender categories reflect the particular gender dynamics of this region of South America.) However, after subsetting the data, we ended up with several variables with no variation, and societies with a high degree of missing data. For this reason, prior to calculating distances between societies, we dropped all variables with less than two levels (that is, one level, or one level and N/A). Subsequently, we created a distance matrix based on the full dataset and used backward elimination to remove those societies with the highest number of non-calculable distances. We repeated this process until we were left with a distance matrix that contained pairwise distances between all remaining societies. For the male-specific subset, this resulted in distances being calculated based on 74 variables for 17 groups. For the female-specific dataset, distances were calculated based on 15 variables for 13 societies.

**Table 2.** Categories of gender-specific cultural practices in the NWA used to subset the sociocultural database.

transmitted from fathers to sons	transmitted from mothers to daughters
Yurupari rites and ritual paraphernalia	non-shamanic crop cultivation
handling of shamanic plants (coca, ayahuasca, tobacco)	food processing and preparation (and associated material culture)
phratric socio-political structure	gathering
house building and woodworking	ceramics
hunting tools and techniques	

### 2.3. Linguistic databases

We consulted written sources to collect data on the grammars of 18 NWA languages belonging to the Tukanoan and Arawakan language families (table 1). We collected data that give us a broad cross-section of language structure, divided into the following topics (for a more detailed description, see the electronic supplementary material):

1. Phonemes and allophones: phonemes and their realizations in different contexts, comparing both the realizations and the characterizations of the contexts.
2. Person: the form and meaning of elements whose interpretation includes grammatical person.
3. Noun classification: the form, (generalized) meaning, and grammatical contexts of noun classification marking strategies.
4. Case marking: the form and meaning of case markers.
5. Tense, aspect, modality, evidentiality marking: the form and meaning of markers that code temporal, aspectual, modal or evidential information.
6. NP syntax: the relative order of elements and morphosyntactic marking patterns of the noun phrase.
7. Clausal syntax: the relative order of elements and morphosyntactic marking patterns of the clause.

These data have all been conceived as inventories that languages may have (e.g. of phoneme-allophone relations, morphemes or syntactic structures). We call the observational units for each of these inventories *constructions*, using the term broadly to mean a recurring syntagmatic pattern of language that pairs a formal realization to an interpretation (form-meaning pair). It is a broad use of the term in the sense that it can be interpreted abstractly, as in the case of phonemes and allophones, for which it can be said that allophones are the formal realization (form) of a phoneme (interpretation, or meaning).

### 2.4. Analysis of sociocultural and linguistic data

After we standardized the databases described in §§ 2.2 and 2.3 using the glottospace R package [78], we calculated the degree of (dis)similarity between the ethnolinguistic groups using Gower's general coefficient of similarity [79]. The resulting distance matrices were used as input to perform Nonmetric Multidimensional Scaling (NMDS) [80]. NMDS results were subsequently plotted in two and three dimensions to explore dissimilarities between ethnolinguistic groups. To assess whether pre-defined sets of ethnolinguistic groups are different from each other to a degree that would be considered statistically significant, we performed overall and pairwise permanova on the raw distance matrices [81]. Data preparation, analysis and visualization were conducted through the workflows implemented in the glottospace R package, which provides wrappers to functions of several other packages, including the cluster package for calculating distances [82], the vegan package [83] for performing NMDS and permanova, and ggplot2 [84] and plotly [85] for visualizing NMDS results.

## 3. Results

### 3.1. Genetics

**Figure 1.**
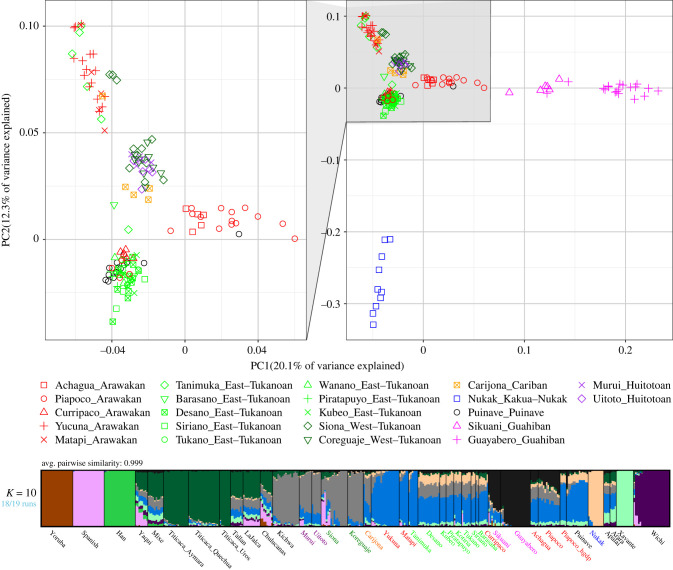
(*a*) PCA depicting PC1 and PC2 from NWA individuals based on 5 72 537 SNPs. (*b*). ADMIXTURE results for the best-fit model of *K* = 10 ancestry components, based on a LD pruned dataset containing 87 297 SNPs.

We used principal component analysis (PCA) to visualize the broad patterns of genetic variation among individuals and groups included in this study (figure 1*a*). Variation in PC1 separates speakers of Guahiban languages on the right from Yukuna and Nukak individuals on the left; Piapoco individuals form a cline towards the Guahiban-speaking groups, reflecting some genetic similarities with them. PC2 separates Nukak individuals at the bottom-left and a cluster of Yukuna, Matapi and Tanimuka individuals on the top-left of the plot. We observe that five out of six Tanimuka individuals cluster together with Yukuna and only one Tanimuka individual appears closer to a cluster formed by the rest of Eastern-Tukanoan (ET) speaking groups, Curripaco, four Piapoco individuals from the HGDP panel [16] and Puinave individuals. These broad patterns of genetic similarities are also observed in the ADMIXTURE analysis. Figure 1*b* shows the results for the K ancestral populations with the lowest cross-validation error (electronic supplementary material, figure S2). We provide the full result (i.e. *K* = 2–14) in the supplementary materials (electronic supplementary material, figure S3). In figure 1*b* we observe that Yukuna, Matapi and Tanimuka are assigned a similar ancestry profile. By contrast, other ET-speaking groups, the language family to which Tanimuka's language belongs, are assigned a mixture of ancestries. However, the ancestry component that is maximized in Yukuna, Matapi, and Tanimuka (blue ancestry) represents on average 46% of the ancestral components assigned to ETs (figure 1*b*).

**Figure 2.**
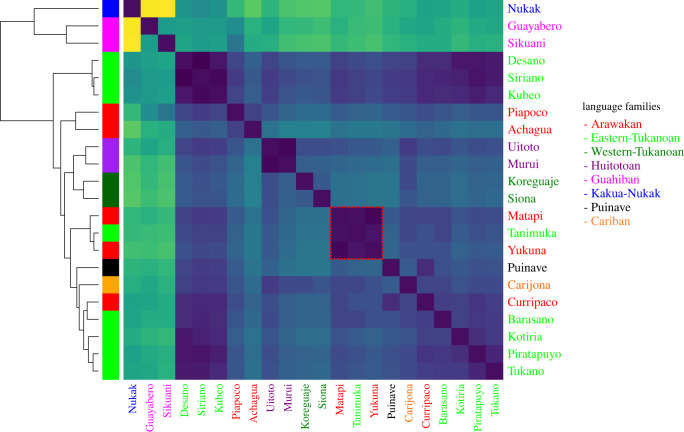
Heatmap of a matrix of pairwise f2-distances. Dendrogram on the left shows a hierarchical clustering of the pairwise distances. Darker colours indicate smaller distances, while light colours indicate larger distances. The cluster formed by Tanimuka, Yukuna and Matapi is highlighted in red.

We used f-statistics to determine genetic affinities between Yukuna, Tanimuka, Matapi and other groups in NWA; for these we excluded individuals that showed more than 10% European- and African-related ancestry (pink and brown components in figure 1*b*). Pairwise f2-distances show the existence of several clusters showing high genetic similarities (figure 2). Tanimuka, Yukuna and Matapi are part of one such cluster (highlighted in red), reflecting the close contact and extensive intermarriage previously documented among these groups (Arias *et al*. [13]). This close genetic similarity is supported by an outgroup-f3-statistic of the form f3(NWA_groups, Tanimuka; Mbuti), which tests which group(s) in NWA shows more genetic affinities to Tanimuka. The highest f3 values were exhibited by Yukuna and Matapi (electronic supplementary material, figure S4).

**Figure 3.**
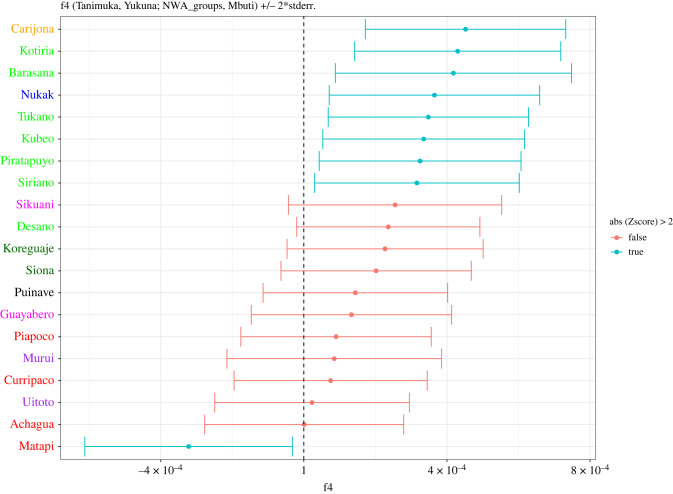
f4-statistic to determine if either Yukuna or Tanimuka share excess ancestry with other NWA groups. Ethnolinguistic names are colour-coded by language family, as indicated in figure 1.

Furthermore, we used a f4-statistic of the form f4(Tanimuka, Yukuna; NWA_group, Mbuti) (figure 3), where NWA_group is each of the comparative groups, tested iteratively in that position, to determine whether Tanimuka and Yukuna differ in how they relate genetically to other groups in the area. This statistic tests whether the four populations are related in a tree-like fashion, based on a measure of the average correlation in allele frequencies between populations [66,86]. This test assumes that Tanimuka and Yukuna form a clade, and that the other clade is formed by an NWA_group and the Central African Mbuti group; if this is the case, this statistic is equal to zero. However, if the statistic is significantly lower than zero, it means that Yukuna shares additional ancestry with an NWA_group that is not shared with Tanimuka. If the statistic is significantly higher than zero, it means that it is Tanimuka that shares additional ancestry with an NWA_group other than Yukuna. Such a deviation from zero indicates a violation of treeness, which is indicative of admixture between groups. This f4-statistic revealed that Matapi shares more ancestry with Yukuna than with Tanimuka, not surprising given that Yukuna and Matapi speak the same language, live side-by-side, and intermarry extensively. Furthermore, we found several other cases in which the tree hypothesis is rejected, since Tanimuka exhibits a significant excess of ancestry sharing with Carijona, Kotiria, Barasana, Nukak, Tukano, Kubeo, Piratatpuyo and Siriano.

Among those groups, Carijona shows the highest positive f4-value. This result is consistent with oral histories of groups from the Apaporis and Mirití-Parana Rivers, who attribute a common origin to the Tanimuka and the Carijona [87,88].

We estimated admixture proportions in Tanimuka using an f4-ratio [16]; based on the results of the f4-statistic (figure 3) we modelled Tanimuka's genetic history as the result of genetic admixture between Yukuna and ET groups, as follows: we tested all ET groups iteratively as contributing ET-related ancestry; Yukuna is the other contributing source; Nukak is used as a reference population with no direct contribution and related to either ET groups or Yukuna, and Mbuti is the outgroup (see electronic supplementary material, table S1, electronic supplementary material, figure S5 for details). We estimated a 40% ET-related ancestry and 60% Yukuna-related ancestry in Tanimuka.

**Figure 4.**
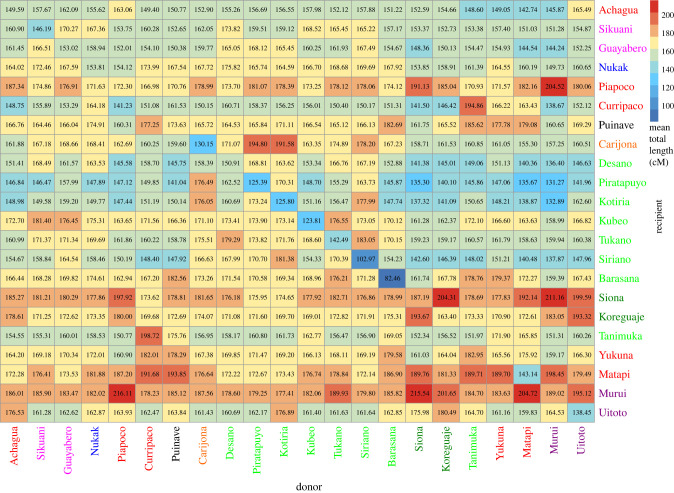
Heatplot of the mean total length in centimorgans (cM) of DNA segments that each recipient group (rows) copies from each donor group (columns). Ethnolinguistic names are colour-coded by language family as indicated in figure 1.

We then investigated whether the observed genetic affinity patterns based on the correlations of allele frequencies were attributable to recent demographic events. For this, we used haplotype-based methods that inform us about events which occurred on the order of tens of generations to a couple of hundred generations up to the present [15,73]. Particularly, we were interested in which groups have contributed ancestries to Tanimuka, Yukuna and Matapi, and whether there are differences in the sources contributing ancestry to them. Figure 4 shows the results of the ancestry painting with ChromoPainter [75], donor populations appear on the *x*-axis and recipient populations on the *y*-axis. Although Yukuna and Matapi appear as donors of ancestry to Tanimuka, it is the Curripaco who contribute more ancestry to Tanimuka, as well as to Matapi and Yukuna. By contrast, ET groups contribute less ancestry to Tanimuka than they do to Yukuna or Matapi. Also, Matapi individuals are recipients of diverse ancestries, since we observe consistent high mean values with multiple groups in NWA, the top donors being Murui, Puinave and Curripaco. However, as has been shown by Hellenthal *et al*. [15], if a source group is genetically relatively similar to a single sampled population, then this population dominates the inferred mixture. If there is no close proxy for the admixing group in the sample (which is especially likely for ancient admixture events or sparsely sampled regions), several donor populations are needed to approximate its pattern of haplotype sharing and then the focal population is automatically a haplotypic mixture of the combined donors, because it is a mixture of the source groups [15].

Moreover, patterns of IBD sharing (electronic supplementary material, figure S6) show that Tanimuka, Yukuna and Matapi share a large number of very long IBD blocks both within and between them, indicating that these three groups have a large number of common ancestors during the last 500 years, a likely consequence of their extensive and continuous genetic admixture through this time period.

#### 3.1.1. Sex-specific genetic patterns

Uniparental data (electronic supplementary material, figure S7*a,b*) shows differences in how Tanimuka and Yukuna relate to each other and to other groups in NWA. For the mtDNA Yukuna seem to have wider connections to other groups than Tanimuka, as Yukuna show more shared and related haplotypes with different ethnolinguistic groups. Tanimuka mtDNA and Y-chromosome haplotypes are mainly shared with or closely related to haplotypes in the Yukuna, with just a few exceptions that show links with haplotypes in the Barasana, Curripaco and Desano and Tukano.

In summary, Tanimuka's main interaction partner is Yukuna, while Yukuna itself seems to have a larger network of interactions on the female-specific domain and on the male-specific domain, Yukuna's Y-chromosome haplotypes are shared or closely related among Yukuna males, and related to Tanimuka's haplotypes.

These observations seem to support a scenario where Yukuna as a group has a larger sphere of influence and intermarriage with a diverse set of ethnolinguistic groups in NWA, while Tanimuka's main interaction partner is Yukuna.

### 3.2. Linguistics

The long-term effects of a language shift are difficult to predict, and as a consequence, they are also difficult to reconstruct on the basis of contemporary data. As famously stated by Thomason & Kaufman ([26]: 14), ‘as far as the strictly linguistic possibilities go, any linguistic feature can be transferred from any language to any other language; and implicational universals that depend solely on linguistic properties are similarly invalid'. Thomason and Kaufman's claim is that social circumstances are the main determinant of the outcomes of language contact. The idea that particular linguistic outcomes can be expected as a result of particular social scenarios has been discussed extensively in the literature (e.g. [89–93], among many others). Nevertheless, social scenarios tend to be intricate and multi-faceted, so any approach that is based on social scenarios is necessarily schematic and simplified.

Our approach to the matter of language shift in linguistics is as follows: on the basis of linguistic distances between constructions (see §2.4), we can assess to what extent Tanimuka and Yukuna are more similar than expected (i.e. not significantly different), and to what extent Tanimuka and Yukuna differ from the corresponding trends in their respective language families. We, therefore, focus on the following three questions:

1. Are Arawakan constructional profiles significantly different from Tukanoan ones?
2. Are Tanimuka and Yukuna significantly different from the respective family profiles?
3. Are Tanimuka and Yukuna significantly different from each other?

**Figure 5.**
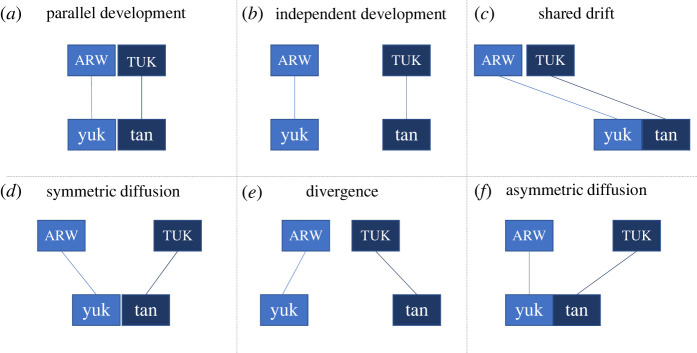
Six generalized patterns and their corresponding likely historical scenarios. Strictly speaking, the three questions spell out 16 possible scenarios, but a number of these can be regarded as variations on these six general patterns.

From these three questions, we can schematically represent six possible patterns, shown in figure 5:^1^

Figure 5 should be read as follows: if the answer to all three questions is ‘no’, this would constitute pattern (a), in which the Arawakan and Tukanoan profiles as well as Yukuna and Tanimuka are all similar. This pattern might be the result of parallel structures between the families, which may be due to coincidence or contact-induced convergence or shift at the proto-language level. The opposite pattern (e), with ‘yes' for all three questions, would be a situation in which Tanimuka and Yukuna have both developed away from their respective family profiles, but also from each other.

The historical scenarios that we focus on in this paper are convergence and language shift. These two scenarios would be compatible with patterns d and f, respectively. Scenario d (symmetric diffusion) can be identified if the Arawakan and Tukanoan profiles are significantly different from each other (Q1 = Y), Tanimuka and Yukuna are different from their respective family profiles (Q2 = Y for both languages),^2^ and Tanimuka and Yukuna themselves are not significantly different from each other (Q3 = N). An asymmetrical diffusion pattern holds if Arawakan and Tukanoan are significantly different (Q1 = Y), one of the languages is different (Q2 = Y) while the other is not (Q2 = N), and Tanimuka and Yukuna are not significantly different (Q3 = N).

**Figure 6.**
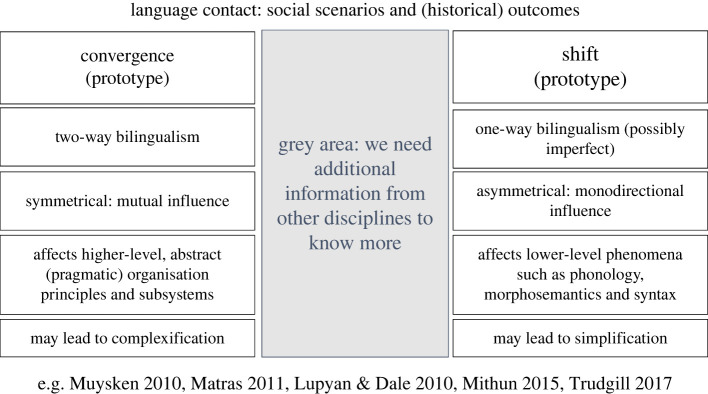
Typical outcomes and characteristics of linguistic convergence and shift.

The typical outcomes and characteristics of convergence and shift are summarized in figure 6, in which the prototypical contact situation is indicated in the top box, and the typical linguistic outcomes in the boxes below.

Note, however, that the connection of (in particular) language shift (figure 6) to pattern f (figure 5*f*) is not straightforward. The presence of pattern f can be a false positive for a historical scenario of shift, and the absence of pattern f can be a false negative. The reason for the former (pattern f is present but not indicative of shift) is because scenario f is also compatible with a situation of language maintenance, in which there is strong inequality in power relations and/or unidirectional bilingualism. Nor does the absence of pattern f exclude a shift scenario.

#### 3.2.1. Linguistic distances of grammatical constructions

**Figure 7.**
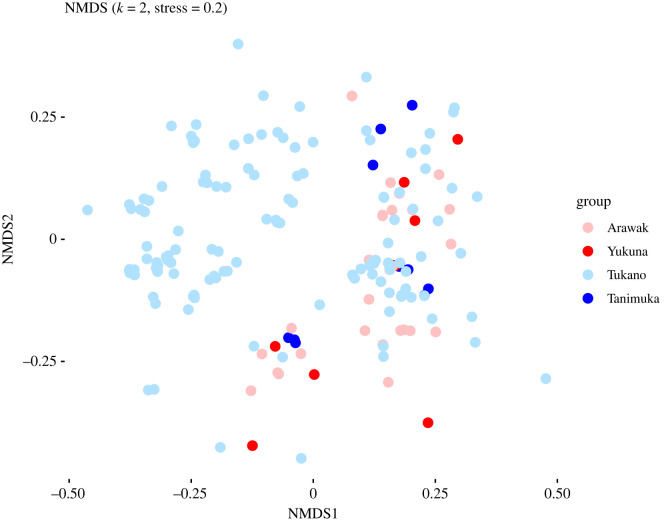
NMDS plot of tense-aspect-modality-evidentiality (TAME) constructions in the languages of the sample.

Of the seven grammatical domains we investigated, three are consistent with a shift scenario. These are classifiers, person markers, and tense-aspect-modality-evidentiality (TAME). The latter shows this pattern most clearly, so we discuss it here. Figure 7 presents an NMDS plot of the TAME constructions. As suggested by the plot, most of the Tanimuka TAME constructions (morphemes) are at the edge of an area that is prototypical of Tukanoan, and in the vicinity of Arawakan constructions.

We tested this visual pattern by applying a permanova test, where we contrasted Arawakan (without Yukuna), Tukanoan (without Tanimuka), Yukuna and Tanimuka. The results are given in table 3:

**Table 3.** Permanova test results for TAME.

group1	group2	*p*-value (adj)	sign (adj)
Tukano	Tanimuka	0.006	**
Tukano	Yukuna	0.012	*
Tukano	Arawak	0.006	**
Tanimuka	Yukuna	0.978	n.s.
Tanimuka	Arawak	0.024	*
Yukuna	Arawak	0.216	n.s.

As can be seen in table 3, the Tukanoan profile is significantly distinct from Arawakan (row 3), and Tanimuka and Yukuna are not significantly different (row 4). At the same time, Yukuna is not significantly different from the Arawakan profile (row 6), whereas Tanimuka is distinct from the Tukanoan profile (row 1). This spells out an asymmetrical pattern, in which Tanimuka has been influenced by Yukuna, but not vice versa.

The other two areas of grammar where we find asymmetrical patterns in which Tanimuka has become Arawakanized, but Yukuna has not become Tukanoanized (or at least much less so) are classifiers and person markers. Nevertheless, the patterns are slightly different for these two datasets (electronic supplementary material, figures S8 and S9 and electronic supplementary material, tables S2 and S3). The results for person markers are similar to those of TAME, except that the difference between Tanimuka and Arawakan is also non-significant. The classifiers show a slightly weaker asymmetrical pattern in that Yukuna is also significantly different from its Arawakan relatives (*p* = 0.012), but where Tanimuka is similar to Arawakan (*p* = 0.45), Yukuna is still significantly different from Tukanoan (*p* = 0.006), and Tanimuka and Yukuna show non-significant differences (*p* = 0.126).

Of the remaining four datasets, two show weak asymmetric patterns. Noun phrase structure is significantly different for all pairs, except for Yukuna and its Arawakan sister languages, but Tanimuka does seem to have become more similar to (though still significantly different from) Yukuna (*p* = 0.042, the highest *p* value of the significantly different pairs, electronic supplementary material, table S4). The phoneme-allophone database shows a pattern in which Tanimuka is both similar to Tukano (*p* = 1) and Arawak (*p* = 0.084), while Yukuna is different from both (*p* = 0.006 for both), suggesting a possible change within Tanimuka toward the Arawakan profile. The last two datasets show different patterns. Case shows parallel structures throughout the two families, including Tanimuka and Yukuna, as none of the groups are significantly different from each other. The only dataset that shows signs of convergence is the dataset on clausal structure, where Yukuna and Tanimuka are not significantly different from Tukanoan and Arawakan, respectively, and from each other (*p* = 0.054, electronic supplementary material, figures S10–S13 and electronic supplementary material, tables S4–S7).

All in all, then, the main signal is asymmetrical, suggesting that Tanimuka has been influenced by Yukuna much more than vice versa. As discussed above in this section, this is compatible with a shift scenario, though not necessarily indicative of it.

### 3.3. Comparative sociocultural data

**Figure 8.**
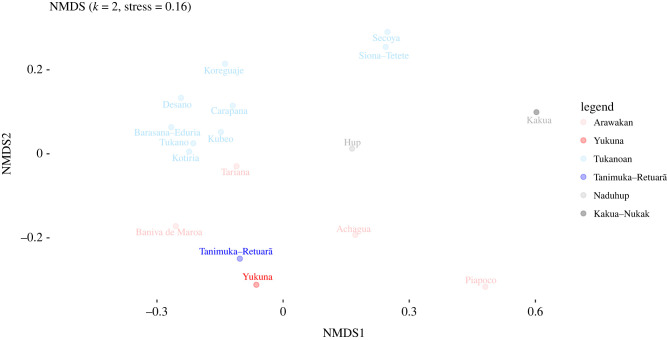
Socio-cultural practices generally transmitted from father to son in the Northwest Amazon.

NMDS plots from the similarity matrices calculated from the complete sociocultural dataset and the gender-based subset appear in figure 8 and electronic supplementary material, figures S14 and S15. In NMDS plots, stress values below 0.1 are considered to give an accurate representation of similarities, values between 0.1 and 0.2 are useful for distinguishing broad-scale patterns, while stress values above 0.2 could be misleading [94]. For this reason, we use the NMDS plots in this paper to explore the data, but we always base our conclusions on PERMANOVA results.

Electronic supplementary material, figure S14 provides a broad overview of the sociocultural patterns in NWA. ET speaking groups form a fairly tight cluster in the top-left quadrant, suggesting a relatively high degree of sociocultural homogeneity among speakers of those languages. By contrast, the Arawakan speaking groups occupy a more diffuse area, indicating a more heterogeneous array of sociocultural practices (a pattern that contrasts starkly to the speakers of ET languages). This is the pattern that we might expect if, as suggested by [95], the Arawakan family spread through the region primarily through language shift and cultural contact rather than primarily through demic migration (a scenario also supported by the genetic data presented in §3.1). Yukuna and Tanimuka-Retuarã are found in an intermediate area that is peripheral but close to both families.

As discussed in §2.2 and table 2 above, we further investigated if subsetting the data on the basis of gendered practices could provide additional insights into the dynamics of population and language contact among NWA societies. The logic behind this decision is two-fold: first, most of the societies in the study area are organized according to exogamous patrilineal descent groups and practise patrilocal post-marital residence. This means that unions are generally formed between spouses from different settlements (traditionally, often malocas), and that women usually move to their husbands' settlements. Men, by contrast, are more likely to stay put. Thus, we would expect cultural practices transmitted among women to be more homogeneous in the region, and cultural practices transmitted among men to be more heterogeneous. Second, there are relatively sharply differentiated social roles associated with each gender in the Vaupes and broader Rio Negro region, at least by the standards of lowland South America (table 2). This would make the gendered pattern in the data more acute.

The NMDS plot in figure 8 shows the distances for sociocultural practices and knowledge that, according to the ethnographic literature from NWA, are generally transmitted from fathers to sons. Here, Tanimuka and Yukuna are quite distinct from ET groups and appear closer to Arawakan-speaking groups, in comparison with the entire dataset (electronic supplementary material, figure S14). We consider this differential pattern as adding further weight to the hypothesis that the Tanimuka paternal lineage came from another group –likely Arawakan– which shifted to an Easten Tukanoan language as they joined the ET social complex through the exchange of women [14,96].

Unfortunately, subsetting the dataset to reflect only cultural practices transmitted from mothers to daughters presented some problems in the analysis. In particular, the high degree of homogeneity in women's cultural practices in the NWA (as defined in table 2) led to a large proportion of variables with a single value. This forced us to discard all but 15 of the variables, making the NMDS plot uninformative (electronic supplementary material, figure S15). However, an informal inspection of the women's sociocultural practices suggests that they are indeed more homogeneous than the men's sociocultural practices: of the 34 women's variables, 19 (56%) were excluded because they had fewer than two levels, while only 38 of 112 (34%) of the male variables were excluded for this reason (we leave a more detailed exploration of these patterns for future analysis). This disparity is what we would expect in a region where women circulate more broadly than men, and it is indeed consistent with the genetic patterns identified by Arias *et al*. [31]. We leave a more detailed exploration of these patterns for future analysis.

## 4. Discussion

In this paper, we have investigated the population and language contact history of Yukuna, Matapi and Tanimuka, ethnolinguistic groups of NWA which coexist in various communities along the Mirití-Parana River and its tributaries, and contextualized them within the larger NWA region. We did this by integrating evidence from linguistics, ethnography, and genetics. Although previous studies have found that these languages have undergone mutual contact-induced linguistic change [5,11,12], so far it has not been clear what the underlying sociocultural and linguistic dynamics of this change might have been. To generate new insights about these processes, we have proposed two historical scenarios on the basis of previous ethnographic and genetic evidence [13,14]. In the first scenario, Tanimuka speakers descend from an Arawakan group related to Yukuna, which later adopted an Eastern Tukanoan (ET) language. The second scenario is that Tanimuka speakers descend from an ET-speaking group, but that extensive contact and intermarriage with Yukuna resulted in notable convergences in their language and culture.

The patterns that emerge from the three lines of evidence are consistent with the intricate and multilayered nature of the interaction between Arawakan and Tukanoan groups. Specifically, we observe that Yukuna, Matapi and Tanimuka have interacted extensively to the point that it is difficult to differentiate between them genetically, or define the boundaries of each ethnolinguistic unit (figures 1 and 2). Regardless, we try to dissect both the chronology and dynamics of these interactions and their outcomes in the genetic, linguistic, and sociocultural patterns of variation.

Genetics and ethnohistorical information can provide insights into the temporal layers of contact among these groups. Haplotype-based methods, in particular, allow us to make inferences about the most recent time scale, on the order of tens of generations before present [15,73]. We can say that Yukuna, Matapi and Tanimuka have extensively intermarried in the last 500 years before present (ybp) (approx. 17 generations), and this can perhaps even be extended to the last 1500 ybp (electronic supplementary material, figure S6).

Carijona and the Barasana seem to have joined this interaction, since they also exhibit a considerable amount of long IBD sharing (greater than 10 cM) with our focal groups. Ethnohistorical accounts as well as the oral traditions of groups from the Mirití-Parana/Apaporis Rivers are consistent with this picture, suggesting that the Tanimuka arrived relatively recently to this area [87], perhaps as a consequence of the colonial era upheavals or more recently during the rubber boom between the 19th and early 20th centuries.

Furthermore, genetic data are also consistent with oral histories and mythology. For instance, according to Yukuna oral histories, the Tanimuka, the Carijona, and white people have the same mythical origin, the jaguar *Jerí*. Similar accounts are found among the Makuna, an ET-speaking group from the Apaporis River, who call the shared jaguar ancestor *Yainakahí*. However, the Tanimuka do not agree, and say instead that *Yainakahí* is only the ancestor of the Carijona and the non-indigenous Colombians [87,88,96]. Although such verbal formulations cannot be read literally as direct accounts of historical events, they can show how indigenous societies have experienced history [97] and the way they have organized time and space to account for the movement, interethnic relationships, and unbalanced contact with ‘whites’. Such interpretations of myth and oral history have been proposed for the Wakuénai from Venezuela [98], relatives to the Curripaco in Colombia and Baniwa in Brazil. However, our sociocultural dataset only shows broad-scale patterns, suggesting a close relationship between Yukuna and Tanimuka and a general similarity among Arawakan and ET-speaking groups from the area (figure 8 and electronic supplementary material, figure S14).

Linguistic data provide evidence of the pervasive contact influences on the Yukuna and Tanimuka languages. We observe signals of both convergence and asymmetrical impact on their languages, in which Tanimuka has been influenced by Yukuna much more than vice versa. This can be seen in figure 6, in which we contrasted *prototypical* shift and *prototypical* convergence scenarios. There are many ways in which actual social scenarios can differ from the ones shown in this figure, and the theoretical opposition between convergence and shift is best regarded as a continuum. In particular, the historical effects of language shift can be all but absent under certain circumstances. Notably, if there is child bilingualism, with full access to L2 (the target language), shift may leave no trace of the original language. In addition, effects may be differential across different parts of the linguistic system. These issues make it difficult to predict outcomes of contact scenarios, and thus to reconstruct social scenarios from contemporary data. In most cases, a reconstruction can suggest a most likely scenario, and even for that, linguistic data need to be combined with data from other disciplines to complete the reconstruction of the social context.

Although there is no way of reconstructing a historical language shift that has left no trace in the linguistic data, we can (again, prototypically) distinguish between maintenance and shift scenarios and thus reduce the risk of a false positive. In their aforementioned influential model of language contact and its outcomes, Thomason and Kaufman [26] make a basic distinction between maintenance and shift scenarios. Each of these scenarios is associated with a different process of contact-induced language change. In a maintenance scenario, influence of one language on the other typically starts in the lexicon, and only affects phonology and grammar after a long, intensive period of contact. Shift, on the other hand, typically affects phonology, syntax and perhaps morphosemantics, but the lexicon is hardly affected.

We, therefore, looked in more detail at the structure of the lexicon from a contact point of view. From a lexicon of 600 words of basic and cultural vocabulary, Yukuna and Tanimuka (also Letuama) share 32 each with a common etymology. This corresponds to 70% of all 46 loans found in the Tanimuka-Letuama lexicon. From this, 9 are exclusively shared between Yukuna and Tanimuka. Among these, 6 are loans from Yukuna into Tanimuka-Letuama, while 3 have an unclear directionality. For the remaining 37 etyma in Tanimuka, 24 come from an Arawakan language other than Yukuna, and 13 are *Wanderwörter* from Tupian, Cariban and unidentified sources. The cognates that are exclusively shared between Yukuna and Tanimuka-Letuama are suggestive of bilateral and historically recent contacts, not of long-term, intensive contact resulting in large amounts of borrowed lexicon (electronic supplementary material, tables S8 and S9). This reduces the likelihood of a maintenance scenario and thus a false positive in cases of asymmetrical diffusion (pattern ‘f’ figure 5).

Similar signals are observed in the genetic data. f4-statistics show that, although Tanimuka is closely related to Yukuna, Tanimuka has additional signals of admixture with Carijona, ET groups and Nukak. However, our genetic analyses suggest that the genetic history of human groups in NWA is very complex, with individuals and groups showing genetic similarities to several other groups, irrespective of linguistic and cultural differences (e.g. figures 2 and 4). Acknowledging these complexities and the assumptions used in the analyses of genetic data, we have attempted to reconstruct the dynamics of genetic contact between Tanimuka and Yukuna. We used f4-ratios [15] to estimate genome-wide admixture proportions in Tanimuka equal to 60% Yukuna-related ancestry and 40% ET-related ancestry (electronic supplementary material, figure S5).

Furthermore, uniparental genetic data suggest that these interactions were different for men and women. We observed that on the male-specific side, Yukuna and Tanimuka's Y-chromosome haplotypes are closely related and different from other Arawakan and ET haplotypes. By contrast, on the maternal side we observed that the Yukuna exhibit more haplotypes and these are related to haplotypes from different ethnolinguistic groups, while Tanimuka's mtDNA haplotypes are mainly shared with Yukuna or closely related to Yukuna haplotypes. This might suggest that Yukuna has been involved in a larger network of interactions with other ethnolinguistic groups in NWA, while the Tanimuka have primarily interacted with the Yukuna. However, this could be due to the large differences in sample size for the uniparental data (table 1). The Y-chromosome network of haplotypes showed larger differences among groups, and shared haplotypes were restricted to within the same ethnolinguistic group, with the exception of one Matapi individual that exhibited a haplotype very frequent among Yukuna individuals. The Y-chromosome data showed bigger differences between groups and shared haplotypes were usually restricted within groups, reflecting less movement of men between groups and consistent with the patrilocal practice among NWA societies. This observation is concordant with the analysis of male-specific sociocultural practices that showed more differences among NWA groups, while Yukuna and Tanimuka are more similar (figure 8).

In conclusion, we cannot clearly reject either of the hypotheses proposed in this study, since the evidence available to us shows signals that are consistent both with language shift and convergence. Therefore, we argue that it is likely that both processes have operated through the long history of contact between Yukuna and Tanimuka and more broadly between Arawakan and Tukanoan-speaking groups in NWA. However, our data clearly show that Tanimuka and Yukuna look genetically more similar to each other than to their respective language family members and that the Tanimuka language has been influenced by Yukuna much more than vice versa, and although both signals are consistent with language shift, it does not rule out convergence with asymmetrical power relations. Genetically, Arawakan-speaking groups do not show strong genetic similarities to each other, with the exception of our sample of Piapoco and Achagua who live side-by-side. Instead, Arawakan groups tend to show genetic affinities with their non-Arawakan geographical neighbours, which is consistent with the idea that Arawakan groups were pivotal in maintaining regional systems of exchange with diverse ethnolinguistic groups, in which multilingualism and intermarriage were common features [95,99,100]. Finally, the patterns that we have observed here could reflect phenomena that were more common across lowland South America [101], where groups that came into intense contact as a consequence of post-colonial disruptions, reductions in population size, geographical displacements, etc. reacted in different ways to create hybrid ethnicities to adapt to the new situations, keeping a collective memory of their mythical origins and maintaining linguistic differences in the process of ethnogenesis.

## Data Availability

The linguistic and sociocultural databases are provided in the electronic supplementary material [102]. Given the sensitive nature of the human genetic data generated in this study, these will not be made publicly available, but deposited to the European Genome-Phenome Archive (EGA; https://ega-archive.org), under accession code EGAS00001006767. Access to the data will be granted by a Data Access Committee upon agreeing the conditions on the Data Access Agreement Form available upon request.

## Footnotes

1 Strictly speaking, the three questions spell out 16 possible scenarios, but a number of these can be regarded as variations on more general patterns.

2 In addition, both Yukuna and Tanimuka would have developed in the direction of the other family, the trait that distinguishes convergence from shared drift, where this would not be the case.
